# A refractive error prediction model for children and adolescents based on ocular biometric parameters

**DOI:** 10.1186/s12886-025-04600-z

**Published:** 2026-01-16

**Authors:** Wei-Jie Zhang, Shu-Li Xie, Yu-Chang Kan, Xin Yu, Xin-Xin Zhang, Xue-Liang Feng, Guang-Hua Zhang

**Affiliations:** 1 Taiyuan Aier Eye Hospital, Taiyuan, 030012 China; 2 Shanxi provincial Eye Hospital, Shanxi Medical University, Taiyuan, 030002 China; 3 Big Data Intelligent Diagnosis and Treatment Industry College, Taiyuan University, Taiyuan, 0300032 China

**Keywords:** Ocular biometric parameters, Deep learning, Myopia prediction, Spherical refraction, Cylindrical refraction

## Abstract

**Objective:**

To develop a predictive model based on ocular biometric parameters and deep learning for studying the progression of refractive errors in children and adolescents.

**Methods:**

This longitudinal observational cohort study included 559 children and adolescents (1,118eyes; 252males, 307females) aged 5–18 years, enrolled at Shanxi provincial Eye Hospital (Taiyuan, China) between 2019 and 2023. Refractive error and ocular biometric parameters were prospectively assessed through serial non-cycloplegic automated refraction and LENSAR 900 biometry, with participants undergoing 2–5 follow-up evaluations at irregular intervals. After data cleaning, preprocessing, and augmentation through truncation techniques, Long Short-Term Memory (LSTM) and Time-aware LSTM (T-LSTM) models were used to predict refractive changes over the next three years. Mean Absolute Error (MAE) and Standard Deviation (SD) were used to evaluate model performance, reported as MAE ± SD.

**Results:**

Baseline MAEs measured 0.40 ± 0.39D (sphere) and 0.30 ± 0.46D (cylinder), exhibiting temporal deterioration over 12 quarters (sphere: 0.35 ± 0.42D→0.52 ± 0.37D; cylinder: 0.20 ± 0.21D→0.33 ± 0.30D).Additional biometric measurements reduced errors across cohorts: with five measurements ≤ 6y achieved 0.11 ± 0.10D (sphere)/0.09 ± 0.10D (cylinder); 10-12y />12y attained 0.18 ± 0.13D/0.10 ± 0.08D. The steepest error reduction occurred in older cohorts (10-12y/>12y), suggesting that fewer measurements achieve lower prediction errors with increasing age.

**Conclusion:**

The deep learning model developed in this study, based on ocular biometric parameters, demonstrated high accuracy and stability in refractive error prediction.

## Introduction

Myopia represents a critical and growing cause of visual impairment globally. Recent comprehensive analyses, such as the Global Burden of Disease (GBD) studies, have meticulously quantified the substantial burden of uncorrected refractive errors across all age groups, with particularly alarming trends observed in children and adolescent [1]. This burden is not uniformly distributed, with East Asia, and China in particular, experiencing an epidemic of unprecedented magnitude. A recent nationwide study in China, tracking trends over 25 years, confirms the severity of this crisis, revealing a high myopia prevalence that has escalated from 1.7% to 6.7% and projecting a continued raise in both myopia and high myopia burdens through 2050 [2].These studies project a continuous rise in prevalence, necessitating effective public health and clinical strategies.

Accurate prediction of individual myopia progression is therefore paramount for implementing timely, personalized interventions that can alter the disease course. Refractive error quantification is a key objective indicator for myopia prevention and monitoring, with longitudinal changes strongly associated with biometric parameters: spherical equivalent (SE), axial length (AL), corneal flat curvature (K1), corneal steep curvature (K2), anterior chamber depth (ACD), and lens thickness (LT) [3, 4]. While ocular biometric-based myopia models parameters show promising prediction [5, 6], their development has been constrained by methodological limitations. Traditional statistical methods, such as linear mixed models, face inherent challenges with longitudinal data, including the “curse of dimensionality” and an inability to capture complex, non-linear temporal dependencies, thereby motivating the adoption of artificial intelligence (AI) [7, 8]. Deep learning (DL), an AI subfield, excels in leveraging such complex, non-linear relationships for ophthalmic tasks, including myopic maculopathy detection [9, 10], and refractive error prediction, with MAEs reported from 1.74 D in ultra-widefield images [11], down to 0.63 D in models identifying vascular correlates of axial length [12]. Despite this, such models are inherently limited by their reliance on static, cross-sectional data which cannot model the dynamic refractive instability of puberty. Consequently, the integration of sequential biometric data using recurrent neural networks represents a critical and unmet need in the field.

We developed an LSTM-based DL framework leveraging longitudinal biometric trajectories (AL, K1/K2, ACD, LT), to predict spherical/cylindrical refraction at clinical timepoints, addressing precision medicine needs in pediatric myopia.

## Method

### Study population and ethical approval

This observation cohort study was conducted at Shanxi Provincial Ophthalmology Hospital, China, and analyzed longitudinal data from 559 children and adolescents recruited between January 2019 and December 2023.The study adhered to the principles of the Declaration of Helsinki and was approved by the Ethics Committee of Shanxi Ophthalmology Hospital (Approval No. SXYYLL-KSSC010). Informed oral consent was obtained from all participants or, where applicable, their parents/legal guardians. This consent was specifically approved by the Ethics Committee. The final cohort included 1,118 eyes (both eyes of 559 participants) with a total of 3,044 examinations. The number of patients who remained under observation and provided data at each sequential visit milestone was as follows: 559 patients (at 2 visits), 270 (at 3 visits), 142 (at 4 visits), 50 (at 5 visits), 18 (at 6 visits), 4 (at 7 visits), and 2 (at 8 visits). (Note: Thus, patients who completed more visits are counted in all preceding visit counts. The total examination count of 3,044 was derived from the complete follow-up record of each individual.)

### Inclusion and exclusion criteria

Participants were children and adolescents aged 5–18 years who were able to cooperate with refractive and biometric testing and willing to undergo follow-up. Exclusion criteria included: amblyopia or unexplained low best-corrected visual acuity (BCVA < 0.1 Log MAR); any ocular or systemic disease that could interfere with examination or follow-up; a history of refractive surgery or presence of high myopia with sight-threatening fundus pathology; and current or prior use of optical or pharmacological myopia control interventions.

### Clinical examination protocol

All measurements were conducted between 08:00 and 14:00 h under standardized lighting conditions by trained personnel following a unified protocol. The protocol included: distance visual acuity test based on the tumbling E log MAR visual acuity chart (Chinese National Standard GB11533-2011), and computerized refraction test with a vision screening instrument (Jia Shi Ying VS550) and an autorefractometer (AR-1, NIDEK). Ocular biometry via LENSAR LS900 (Haag-Streit): AL, K1, K2, central corneal thickness (CCT), white-to-white distance (WTW), ACD, and LT.

### Definition of a training sample

A training sample was an input-output pair constructed from an eye ‘s longitudinal visits. The input sequence consisted of previous visits, each providing the seven biometric parameters (AL, K1, K2, CCT, WTW, ACD, LT), spherical and cylindrical refractive error, and the patient’s age at that visit, with sex as a static feature for the entire sequence. The prediction horizon (ΔT) was 3–36 months from the last visit in the input sequence to the future target visit. The output was the spherical and cylindrical refractive error values at the target visit.

### Sample size consideration

Adequate sample size is critical to prevent overfitting in predictive modeling. Our cohort of 1,118 independent eyes amply satisfies the common heuristic of 10–20 samples per predictor variable, given our model uses ~ 10 core features per time step. To ensure precise performance estimation, a supportive power analysis was conducted for our primary outcome (MAE of spherical equivalent). Based on pilot data variability (SD ≈ 0.80 D) and a clinically relevant difference of 0.25 D in MAE, an independent test set of > 100 eyes provide sufficient statistical precision (80% power, α = 0.05). Our data partitioning strategy (Section “Data augmentation, partitioning, and stratified analysis framework”) ensures this requirement is met.

### Data augmentation, partitioning, and stratified analysis framework

**Fig. 1:**
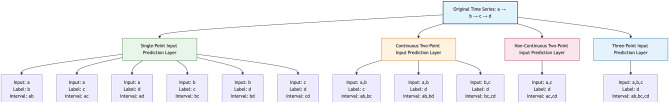
Schematic illustration of the pairwise truncation technique for data augmentation. The original longitudinal data from a single patient is systematically truncated to generate multiple training samples, Architecture of the high-point input prediction layer, demonstrating how individual time points serve as inputs to predict subsequent measurements across the temporal sequence

To prevent data leakage and maximize data utility, we implemented a rigorous pipeline. First, a patient-level split was performed: all 559patients (and thus their 1,118 eyes) were randomly assigned to training (80%), validation (10%), and test (10%) sets. This ensuring all visits from both eyes of any individual patient remained within the same data subset, thereby eliminating the risk of cross-subject information leakage. Second, temporal augmentation was applied exclusively within the fixed training set using a pairwise truncation strategy Fig. 1(implemented with Pandas v1.1.5). For each eye in the training ser, all chronologically ordered visit pairs within a 3–36-month prediction horizon was generated, expanding the raw visits in the training cohort into 14,812 unique temporal samples. across the cohort. The validation and test sets contained only the original, unaugmented sequential data.

The model was designed to predict spherical and cylindrical refractive error at future visits (up to 12 quarters ahead). To ensure robust and clinically interpretable analysis, the study employed a dual stratification and pre-specified subgroup handling approach:

(1) Stratification Variables: Data were stratified by (1) the number of historical visits (2–5 visits) and (2) baseline age cohort (≤ 6, 7–9, 10–12, > 12 years) to account for developmental variations.

(2) Subgroup Robustness Rule: Prior to analysis, a minimum subgroup size of *n* ≥ 5 was required for performance reporting. Subgroups below this threshold (e.g., ‘>12 years’ at the 5th examination, n = 1) were merged with the adjacent age group (‘10–12 years’).

Key Notes: (1) Final analysis was restricted to eyes with 2–5 visits due to limited samples with higher visit counts. (2) The augmented sample counts in stratified results derive from processing longitudinal sequences, not from an increased number of independent subjects. (3) This framework guarantees no data leakage across training, validation, and test sets.

### Model architecture and training

**Fig. 2:**
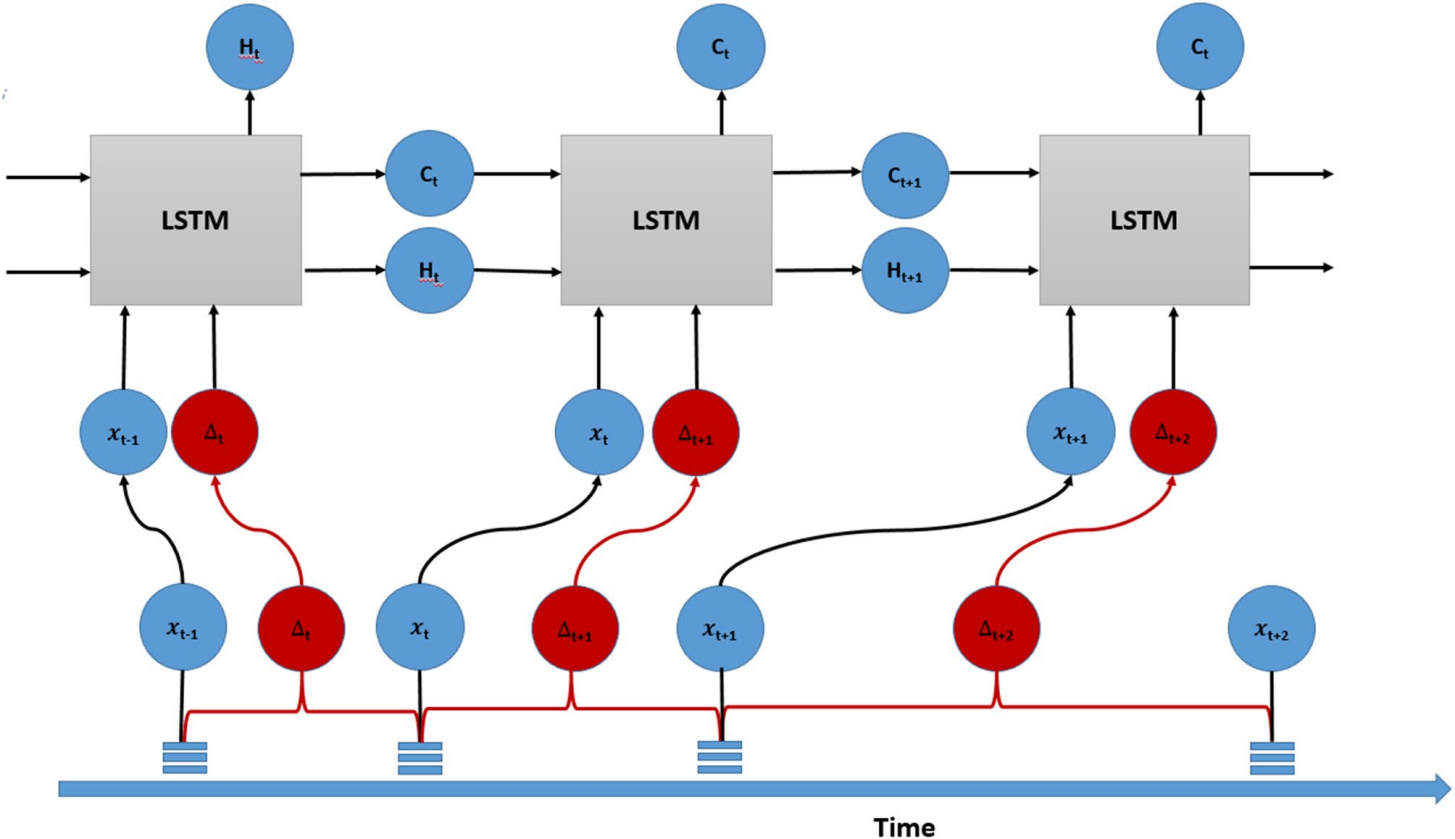
Schematic architecture of the time-aware LSTM model for longitudinal ocular data analysis. The proposed LSTM architecture incorporates sequential time-aware units to process irregular ophthalmic measurements. Each LSTM layer (blue blocks) maintains a cell state (C, horizontal line) and hidden state (H, vertical arrow) to capture temporal dependencies. The temporal dimension (bottom axis) accommodates variable follow-up intervals through adaptive decay mechanisms. LSTM units optimized with temporal decay weights(δ) to modulate historical information retention. Time axis scaling: 1 unit = 3month

We developed a Time-aware LSTM (T-LSTM) Fig. 2 to address the limitation of standard LSTMs in modeling clinical time series with irregular observation intervals. The architecture incorporates two core mechanisms: (1) A Time-aware Memory Decay that weights historical information by the elapsed time (ΔT) between visits, and (2) An Adaptive Interval Learner that captures interval-dependent progression patterns.

The model was implemented in TensorFlow (v2.8). Hyperparameters (including learning rate, hidden units, dropout rate, and Huber loss δ) were optimized via 100 cycles of Bayesian optimization over defined search spaces to minimize validation MAE. The optimal configuration included a learning rate of 0.001, 128 hidden units, and a batch size of 128. Training used the Adam optimizer and proceeded for up to 1,000 epochs with early stopping. The model processes longitudinal biometric parameters and their time intervals to simultaneously predict spherical and cylindrical refractive errors at future time points.

### Benchmarking and evaluation

The T-LSTM was rigorously benchmarked against a suite of baseline models on identical data splits (Section “Data augmentation, partitioning, and stratified analysis framework”), including: a Standard LSTM, Random Forest, Gradient Boosting Decision Trees (GBDT), and a Linear Mixed-Effects (LME) model Table 2.

Performance was primarily evaluated using the Mean Absolute Error (MAE ± SD) for predicting spherical and cylindrical refraction. Differences between the T-LSTM and each baseline were assessed for statistical significance using paired Wilcoxon signed-rank tests on the test set errors.

**Fig. 3:**
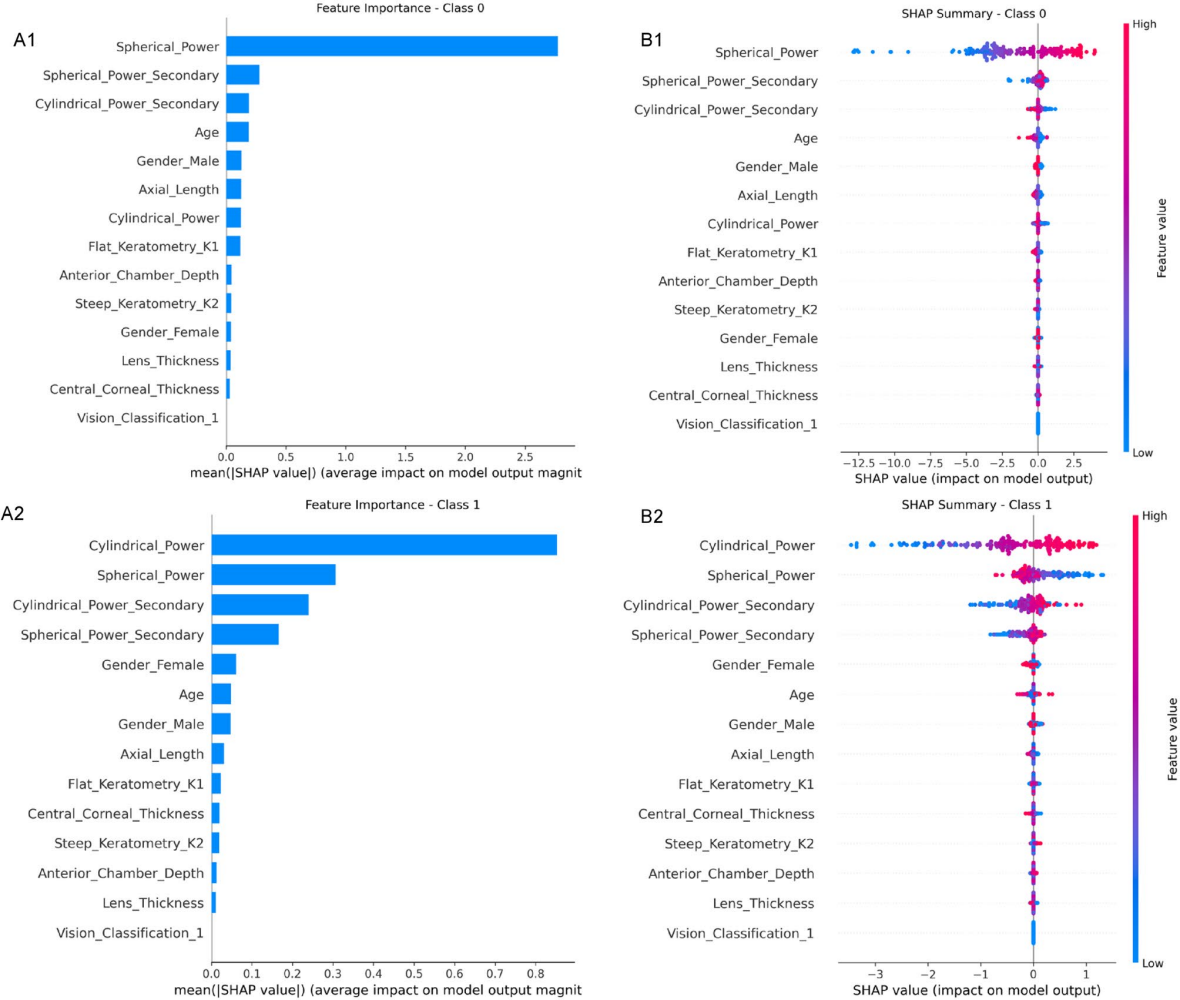
SHAP analysis of the refractive error prediction model. (**A**) Feature importance ranking based on mean absolute SHAP values for predicting spherical power, (Class 0) and cylindrical power, (Class 1)., (**B**) SHAP summary plots showing the directional impact of features on model predictions. Red indicates higher feature values, which are associated with increased predicted refractive error

To interpret the model, we conducted a feature ablation study (quantifying performance degradation upon removing feature groups) and computed SHapley Additive exPlanations (SHAP) values using Tree Explainer on the Random Forest baseline, visualizing how individual features influence predictions (Fig. 3).

## Results

### Cohort characteristics

A total of 559 participants (252 males and 307 females) were included in the study, with ages ranging from 5 to 18 years (median: 8.17 years). The cohort exhibited wide variability in ocular parameters(Table 1.) Spherical power ranged from − 14.25 D to 10.50 D, cylindrical power ranged from − 6.75 D to 4.00 D, and spherical equivalent ranged from − 14.88 D to 10.12 D. Key biometric measurements, including AL, K1, K2, CCT, ACD, LT, also showed substantial ranges, reflecting the diversity of the study population.

**Table 1:** Demographic characteristics and ocular biometric parameters of the pediatric cohort

Characteristic	Statistics
Participants(n)	559(1,118 eye)
Gender	Male: 252(45.1%) Female: 307(54.9%)
Age(years)	8.17 ± 2.15, [5,18]
Refractive Parameters	
Spherical power (D)	-0.36, [-14.25,10.50]
Cylindrical power (D)	-0.75, [-6.75,4.00]
Spherical equivalent (D)	-0.74, [-14.88,10.12]
Biometric parameters	
Axial length (mm)	23.39, [19.01,28.36]
Anterior chamber depth (mm)	3.06, [1.88,3.84]
Lens thickness (mm)	3.52, [2.67,4.77]
Central corneal thickness (um)	548.15, [449,688]
Flat keratometry (K1, D)	42.54, [37.47,47.95]
Steep keratometry (K2, D)	44.26, [39.62,51.47]

Data are presented as n (%) or median [range]. D = diopters; mm = millimeters; um = micrometers

### Overall model performance and benchmark comparison

The proposed T-LSTM model achieved the lowest prediction error for spherical equivalent refraction (Table 2), with a mean absolute error (MAE) of 0.40 ± 0.33 D (95% CI: 0.37–0.44 D). As detailed in Table 2, it demonstrated statistically significant superior performance over each baseline model (all *p* < 0.05). The performance ranking by spherical MAE was T-LSTM (0.40 D) > GBDT (0.51 D) > standard LSTM (0.52 D) > Random Forest (0.53 D) > Linear Mixed Model (0.65 D). For cylindrical power prediction, the T-LSTM also yielded the lowest error among all models, with an MAE of 0.30 ± 0.46 D.

**Table 2:** Comparative performance of prediction models for spherical refractive error

Model	Spherical MAE (D)	SD of MAE (D)	95% CI of MAE	vs.-LSTM (*p*-value)
T-LSTM(Proposed)	0.40	0.39	[0.37, 0.44]	-
LSTM	0.52	0.36	[0.49, 0.56]	0.012
Random Forest	0.53	0.41	[0.49, 0.58]	< 0.001
GBDT	0.51	0.40	[0.48, 0.55]	0.005
Linear Mixed Model	0.65	0.49	[0.60, 0.70]	< 0.001

CI = Confidence Interval. All values are presented in diopters (D) and rounded to two decimal places. P-values are derived from paired Wilcoxon signed-rank tests comparing each baseline model against the proposed T-LSTM model

### Feature importance and model interpretability

Feature Ablation Analysis. Systematic feature removal validated the model’s design and key drivers. Baseline refraction was the most important predictor: removing spherical and cylindrical refractive error increased the spherical MAE by 59% (from 0.44 D to 0.70 D). When refraction data were excluded, axial length became critical; its removal increased MAE by 121% (from 0.70 D to 1.55 D). Ablating the time-aware mechanism severely degraded performance, yielding an MAE of 0.72 D—higher than that of a standard LSTM (0.48 D)—confirming that modeling irregular intervals is essential.

SHAP Analysis and Temporal Interactions. SHAP analysis (Fig. 3) identified historical spherical power as the strongest predictor of future myopia, followed by axial length and corneal curvature. Greater baseline myopia and longer axial length consistently predicted greater future myopia. A significant interaction was found between patient age and the number of historical visits (β = -0.15, *p* = 0.023): while more visits improved accuracy overall, younger children benefited more. Baseline spherical equivalent was independently associated with MAE (*p* < 0.001). No significant interaction was observed for sex (*p* = 0.52).

### Prediction accuracy as a function of time and data availability

Prediction accuracy demonstrated clear dependencies on both the forecast horizon and the availability of longitudinal data.

#### Influence of prediction horizon

**Fig. 4:**
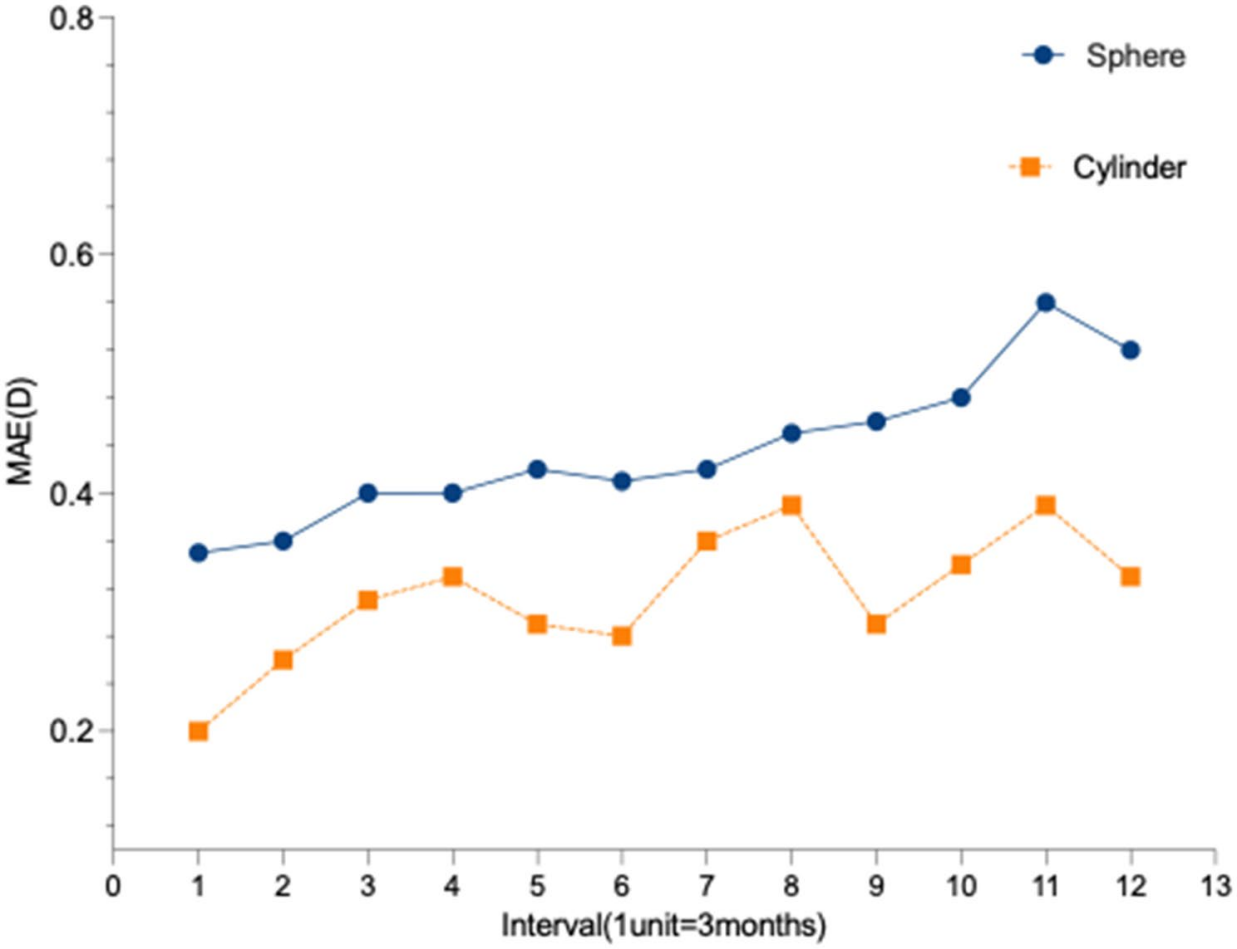
Temporal Evolution of Prediction Errors for Spherical and Cylindrical Powers Over 36 Months. Line chart showing the mean absolute error (MAE, in Diopters) for spherical power (blue line) and cylindrical power (orange line) predictions. Data are presented across 12 consecutive quarters (3-month intervals). The x-axis represents time in quarters (1 unit = 3 months), and the y-axis shows the MAE

Prediction error increased with longer forecast horizons (Fig. 4).For spherical power, the MAE rose from 0.35 ± 0.42 D (quarter 1) to 0.52 ± 0.37 D (quarter 12). A parallel trend was observed for cylindrical power, with MAE increasing from 0.20 ± 0.21 D to 0.33 ± 0.30 D over the same period.

#### Impact of historical measurement count

Model accuracy improved substantially with a greater number of historical biometric measurements. The MAE for both spherical and cylindrical predictions decreased progressively as the number of measurements increased from two to five. With only two measurements, spherical MAE was 0.48 ± 0.43 D, which was reduced to 0.17 ± 0.15 D when five measurements were available. Cylindrical MAE showed a similar reduction from 0.36 ± 0.48 D to 0.16 ± 0.31 D.

#### Moderating effect of age

**Fig. 5:**
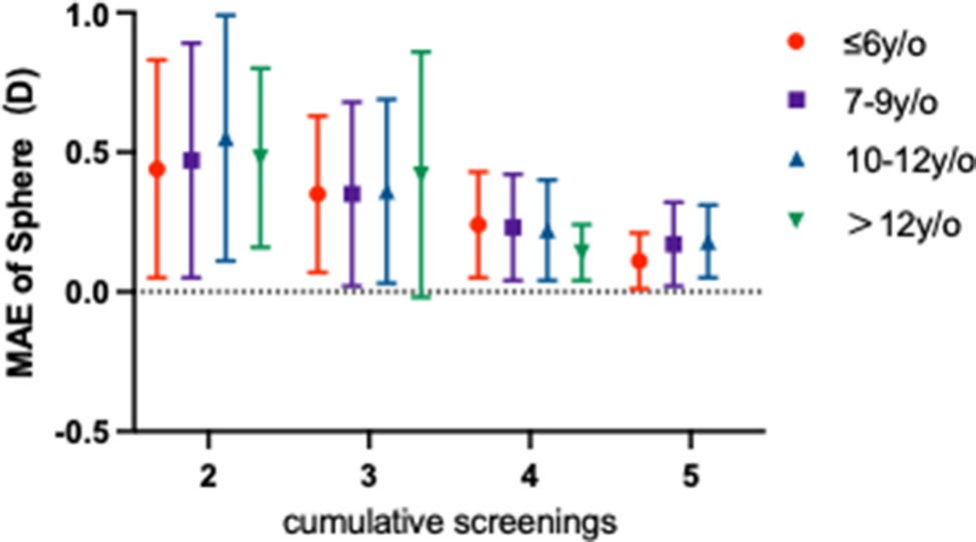
Mean absolute error in spherical power prediction across sequential visits, stratified by age. Grouped bar chart showing the mean absolute error (MAE, in Diopters) of spherical power predictions across four age cohorts (≤ 6, 7–9, 10–12, and > 12 years) from the second to the fifth consecutive visit. The x-axis indicates the visit number (Visit Index), representing the cumulative count of examinations performed. Error bars represent the standard deviation of the MAE. *Per the pre-specified analysis criterion, data from the ‘>12 years’ subgroup at Visit 5 (*n* = 1) was pooled with the ‘10–12 years’ group for analysis

**Fig. 6:**
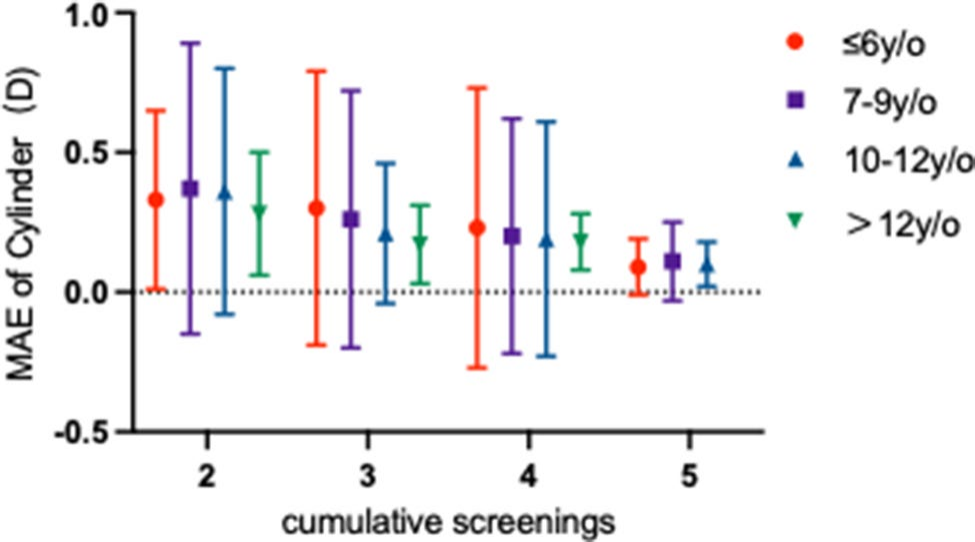
Mean absolute error in cylindrical power prediction across sequential visits, Stratified by Age. Grouped bar chart showing the mean absolute error (MAE, in Diopters) of cylindrical power predictions across four age cohorts (≤ 6, 7–9, 10–12, and > 12 years) from the second to the fifth consecutive visit. The x-axis indicates the visit number (Visit Index), representing the cumulative count of examinations performed. Error bars represent the standard deviation of the MAE. *Per the pre-specified analysis criterion, data from the ‘>12 years’ subgroup at Visit 5 (*n* = 1) was pooled with the ‘10–12 years’ group for analysis

*Note: The > 12 years subgroup at the fifth examination contained only one sample, which exhibited an exceptionally low MAE (0.09 D). Following our pre-specified subgroup robustness criterion (*n* ≥ 5), this data point was consolidated with the 10–12 years group. This process resulted in a negligible change to the aggregated MAE (from 0.180 D to 0.176 D), supporting the robustness of the primary findings that both more historical measurements and older age enhance prediction accuracy.

Age was suggestively associated with predictive efficiency. For a given number of measurements, older age groups tended to achieve lower prediction errors. With two measurements, the spherical MAE in the > 12 years group (0.48 ± 0.32 D) was already relatively low despite a smaller sample size. This trend became more pronounced with three or four measurements, where the 10–12 and > 12 years groups showed the greatest error reduction (Figs. 5 and 6). The lowest errors were consistently observed with five measurements across all age groups.

## Discussion

In this study, we developed and validated a time-aware deep learning model capable of predicting future spherical and cylindrical refractive errors in children and adolescents using sequential ocular biometric data. The model achieved a MAE of 0.40 ± 0.39 D for spherical power and 0.30 ± 0.46 D for cylindrical power. Performance was influenced by three key factors: longer forecast horizons increased prediction error; more historical examinations significantly improved accuracy; and older age was associated with lower error for a given number of measurements, suggesting an age-related stabilization in ocular growth.

Our results confirm that predictive accuracy is governed by two key temporal dimensions: the forecast horizon and the availability of historical data. Prediction error increased significantly with longer horizons, reflecting the growing uncertainty in projecting ocular growth further into the future. Conversely, model precision improved dramatically with a greater number of sequential biometric measurements. This dose-response relationship underscores a critical clinical implication: regular, sequential monitoring is essential for generating reliable, individualized forecasts, supporting the practical value of integrating such predictive tools into ongoing management plans where each new examination refines the future trajectory.

The physiological basis of refractive development lies in the dynamic, visually guided remodeling of ocular components—a process known as emmetropization. In this coordinated growth, AL plays a central but not isolated role [13, 14],. As AL elongates, it interacts with CC [15, 16], CCT [16] ,ACD [17, 18]., and LT [19, 20] to maintain optical focus on the retina. Our model’s strong predictive accuracy stems from its ability to integrate these interacting parameters into a multivariate temporal framework, capturing the non-uniform and compensatory relationships that define refractive progression.

Our feature importance analyses further clarify the relative contribution of key predictors. Spherical power was the strongest individual predictor, followed by AL and CC—consistent with their established roles in myopia development. Notably, the model also captured meaningful interactions, such as the synergistic effect of younger age and greater visit frequency on prediction accuracy. This suggests that in younger children, who exhibit more dynamic ocular growth, more frequent monitoring yields disproportionately greater gains in predictive precision. These insights not only validate the physiological plausibility of the model but also offer practical guidance for structuring clinical monitoring schedules.

Our time-aware sequential model demonstrates robust predictive performance, deriving its strength from the combination of longitudinal data and a specialized architectural design. This represents a significant progression beyond traditional models based on cross-sectional data. For example, the foundational multivariable model by Zadnik et al., which relied on baseline biometry alone, reported a mean absolute error (MAE) of approximately 0.82 D for spherical equivalent [7]. Even more recent machine-learning algorithms that integrate multiple biometric parameters, such as the hybrid model by Barraza-Bernal et al., remain constrained by static inputs, yielding a prediction error range of -0.91 to 0.80 D [21]. This underscores a fundamental limitation of snapshot data in modeling the dynamic process of refractive progression. Studies using alternative data modalities reveal different compromises. Varadarajan et al. achieved an MAE of 0.56 D for spherical equivalent by analyzing features from adult fundus photographs [12]. Our work directly contributes to the emerging paradigm of time-aware deep learning for refractive prediction, as recently advanced by Varošanec et al.[22]. Their modified time-aware LSTM, utilizing longitudinal data with cycloplegic refraction as a gold standard, demonstrated exceptional accuracy (MAE of 0.10 ± 0.15 D). This powerfully validates the high potential of this architectural approach. Our study provides critical complementary evidence in a broader clinical context. We confirm that a time-aware LSTM can achieve strong predictive accuracy (spherical MAE of 0.40 D) using sequential biometric parameters derived from routine non-cycloplegic examinations—data that is far more accessible for standard clinical practice and large-scale screening. Moreover, by systematically evaluating performance across refractive subgroups, we help define the model’s clinical scope of applicability, particularly noting its reduced accuracy in moderate-to-high myopia, a pattern consistent with the findings of Varošanec et al.

The technical superiority of our T-LSTM arises from its explicit design to handle the irregular, longitudinal structure of real-world clinical visits. Its core time-aware mechanism yielded a statistically significant improvement in accuracy over a standard LSTM (MAE: 0.40 D vs. 0.52 D; *p* = 0.012). The model also demonstrated decisive advantages over non-sequential models such as Random Forest and GBDT (*p* < 0.01), underscoring the necessity of capturing temporal dependencies in refractive progression modeling. Furthermore, its substantial outperformance of a Linear Mixed Model (MAE: 0.40 D vs. 0.65 D) highlights its enhanced capability to represent the complex, non-linear dynamics underlying myopia development.

The clinical applicability of the model is further informed by its performance across refractive subgroups. While it maintained high accuracy in non-myopic and low-myopic eyes, predictive performance diminished in moderate and high myopia. This pattern suggests that the model is currently best suited for early-stage myopia progression prediction, consistent with the more variable and complex scleral and optical remodeling characteristics known to accompany advanced myopia. These findings help delineate the model’s appropriate clinical scope and highlight the need for future development of specialized predictors for higher myopic ranges. Several limitations should be acknowledged. First, the model was trained on data from a hospital-based Chinese pediatric cohort and did not include participants undergoing active myopia control interventions such as orthokeratology [23] or atropine [24]; thus, its generalizability to treated populations requires further validation. Second, the use of non-cycloplegic refraction may have introduced systematic measurement bias, particularly in younger children. A substantial portion of our cohort consisted of children under 12 years of age, who typically exhibit high accommodative tone. Non-cycloplegic measurements may overestimate myopia (or underestimate hyperopia), with a mean difference post-cycloplegia reported to exceed 1.00 D [25], especially in hyperopic children or those with less accommodative lag [26]. Moreover, baseline accommodative tone itself has been associated with refractive progression [27]. Consequently, the absence of cycloplegic data may affect trajectory prediction accuracy, warranting future validation with cycloplegic protocols. Finally, as noted earlier, the model showed reduced performance in moderate and high myopia subgroups, indicating that its current utility is greatest in pre-myopia and low myopia. Future research involving larger, multi-ethnic cohorts and interventional data will be essential to enhance robustness and broaden applicability.

It is important to emphasize that subgroup analyses—stratified by age and refractive error category—are exploratory in nature. This study was not prospectively powered to detect effects within these subgroups, and observed significant interactions (e.g., age × visit count, *p* = 0.019) should be interpreted with caution. Potential Type I error from multiple comparisons or Type II error due to limited stratum-specific sample sizes necessitate validation in future studies designed a priori for subgroup hypotheses. Thus, the reported patterns (e.g., greater monitoring benefit in younger children or attenuated performance in high myopia) are considered hypothesis-generating and require confirmation in larger prospective cohorts.

## Conclusion

In summary, we developed a Time-aware LSTM model that accurately predicts future spherical and cylindrical refractive errors by learning from sequences of routine ocular biometric measurements. Crucially, the model is designed to directly handle the irregular timing of real-world clinical visits, transforming longitudinal data into a practical tool for personalized myopia management. Our analysis demonstrates that predictive accuracy improves with more historical examinations and varies with patient age, highlighting the importance of tailored monitoring strategies. While the model shows greatest reliability in pre-myopia and low myopia, its performance delineates a clear scope for clinical application. Future integration with data from wearable devices and interventional cohorts will further enhance its utility and precision, paving the way for more dynamic and individualized approaches to myopia control.

## Data Availability

The datasets generated and/or analyzed during this study (longitudinal biometric and refractive measurements) are not publicly available due to restrictions concerning patient privacy protections for minors under Chinese regulations and ethical governance by the Ethics Committee of Shanxi Ophthalmology Hospital (Approval No. SXYYLL-KSSC010). De-identified data are available from the corresponding author upon reasonable request, subject to institutional review.
